# Between Indifference and the Regimes of Truth. An Essay on Fundamentalism, Tolerance and Hypocrisy

**DOI:** 10.1007/s11406-015-9596-4

**Published:** 2015-04-30

**Authors:** Theo W. A. de Wit

**Affiliations:** Tilburg School for Theology (TST), Tilburg University, Tilburg, The Netherlands

**Keywords:** (In)tolerance, Difference, Truth, Political and existential fundamentalism, Hypocrisy, Subjectivism, Exclusion

## Abstract

There are two basic positions where tolerance as political strategy and moral viewpoint is rejected or made redundant. We are hostile to tolerance when we hold that we are defending an objective truth—religious or secular—which should also be defended and maintained by means of political and legal power. And tolerance become superfluous also when the affirmation of plurality becomes total, and tolerance identical to a vive la difference. As recent developments in my own country—the Netherlands—have demonstrated, the political outcome of this last position is remarkably enough not necessarily an all-inclusive relativistic tolerance. It may just as well be one of intolerance towards ‘believers’ of all kinds, in short: tolerance becomes polemical and belligerent. Turning to religious fundamentalism or ultra-orthodoxy could then become a possible (extreme) reaction to this relativistic and subjectivist position, as demonstrated in Isaac Bashevis Singer’s novel The Penitent. Between these two positions of hostility or indifference towards tolerance, we can situate that democratic attitude which may rightly be called ‘tolerance’. As ethical position, the tolerant citizen accepts the democratic disjunction between my (private) truth and the symmetrical justice between citizens. As political strategy, a tolerant democratic regime is based upon a political act of exclusion of what I will here call ‘political fundamentalism’.

‘I won’t have much to say about the arrangements that get ruled out entirely—the monolithic or totalitarian political regimes’, the American political philosopher Michael Walzer informs us in the Introduction to his *On Toleration* (1997). This narrowing of his theme has not remained unchallenged. If Walzer is only interested in regimes and discourses already becharmed by tolerance, his compatriot Stanley Fish wonders, is there still a problem? Should not tolerance as political strategy and moral viewpoint be proof against precisely the troublesome cases, ‘those forms of thought indifferent or hostile to the tolerance that is his subject?’ (Fish 1999)

‘Toleration makes difference possible’, says Walzer, ‘and difference makes toleration necessary’.^1^ There are indeed two basic positions where tolerance as political strategy and moral viewpoint is rejected or made redundant. We are hostile to tolerance when we hold that we are defending an objective truth—possibly even still to be partially realized in historical-philosophical terms—which should also be defended and maintained officially, by means of political power. What Walzer calls ‘difference’ in this instance represents a denial or deviation from objective truth, or an obstacle to historic necessity. Whoever denies this truth ought to be punished as ‘dissident’, re-educated, or should merely be tolerated in the way we temporarily tolerate irregularities until the i‘s are crossed and the t’s are dotted again.

We nowadays tend to associate ‘fundamentalism’ and ‘theocracy’ with religious regimes. Yet much of the twentieth century saw a grim confrontation with another monolithic truth-regime, the scientistic or rationalistic one of ‘concrete socialism’. Also here a truth was elevated to the throne of power, and not only were those who refused to profess this truth (‘dissidents’) imprisoned or exiled, the regime also exacted a price of ubiquitous hypocrisy.^2^ Here intolerance is not the result of an eruption of the irrational such as is often associated with religious fanaticism and militant devotion, but precisely of the ‘illusion of the omnipotence of reason’ (Finkielkraut 1995), of assuming that the method and rigour of the natural sciences could simply be transposed to the domains of the social and political. In short, removing the religious foundation from a society apparently does not suffice. And hypocrisy was already the by-product of those societies which at the beginning of the modern era continued the strive towards religious homogeneity. (I) In conclusion to my essay I will show that the establishment of a truth-regime has continued to remain a temptation to modern democracies confronted by an increasing social and cultural diversity.

However, tolerance become superfluous or even taboo not only when *difference* is regarded as an inadmissible denial of the truth, but also when the affirmation of plurality becomes total, and tolerance identical to a *vive la difference!* Also when all moral and religious truths become ‘subjective’, like luxuries in which one may or may not choose to invest, does tolerance lose its object and meaning. As recent developments in my own country—the Netherlands—have demonstrated, the political outcome of this is remarkably enough not necessarily an all-inclusive relativistic tolerance. It may just as well be one of intolerance towards ‘believers’ of all kinds, that is, anyone unable to hold tolerance itself as the highest good. Tolerance then becomes polemical and belligerent. (II)

Turning to religious fundamentalism or ultra-orthodoxy could then become a possible (extreme) reaction to this relativistic and subjectivistic position, as demonstrated in Isaac Bashevis Singer’s *The Penitent*, a novel which traces the moral-psychological logic of a conversion to ultra-orthodox Judaism. (III)

Between these two positions of hostility or indifference towards tolerance, we are eventually able to situate that democratic attitude which may rightly be called ‘tolerance’. As ethical position, the tolerant citizen accepts the unavoidable ‘narcissistic wounding’ given with democracy’s disjunction between my (private) truth and the symmetrical justice between citizens, the *equa libertas* of Ancient Rome. As political strategy, a tolerant democratic regime is based upon a political deed of exclusion of what I will here call ‘political fundamentalism’—in distinction to ‘existential fundamentalism’, which in no way needs to be at odds with democratic tolerance. (IV)

## I ‘Thinking One Thing and Saying Something Else’: Hypocrisy as Modern Problem

Even if freedom of opinion could be suppressed, wrote Spinoza in the famous final chapter of his *Theological-Political Treatise* in the context of Amsterdam of more than three centuries ago*,* then it will certainly never happen that the people ‘think only what the authorities want, and thus it would necessary follow that man would be continually thinking one thing and saying something else. This would undermine the trust which is the first essential of a state; detestable flattery and deceit would flourish, giving rise to intrigues and destroying every kind of honest behaviour (de Spinoza 2008).’ The price of a religious state-truth is therefore the ‘doubling’ of truth.

One of his predecessors, the great seventeenth century theorist Thomas Hobbes, indeed had accepted a difference between public ‘confession’ (a minimal religious truth decided and prescript by the state) and ‘faith’ (the inner conviction of the citizen). Spinoza’s hope was that by permitting explicitly what was unavoidable anyway (that people think and judge the way they want) the state could benefit under the provision of the freedom of judgment, because the best ideas could flourish. His example was Amsterdam, the city ‘which enjoys the fruits of this liberty, with its great growth being the admiration of all nations’.^3^

But in his study on Hobbes, the twentieth-century political thinker Carl Schmitt makes another evaluation of the dynamic inherent to the freedom of religion and judgement. He points out the moment where the ‘decisionism’ of the Hobbesian absolute monarchy becomes an object of ridicule, and thereby already loses its theological legitimacy. For example, in his *Leviathan* Hobbes wrestled with the then-important problem of the miracle, in other words, the immediate intervention of God in the natural realm. Whether a given event constitutes a miracle, remains for the sovereign to decide, says Hobbes, but, in his heart of hearts the citizen remains free to believe in the miracle or not: after all, thinking is free (Hobbes 1651). ‘Here nothing is true, here all is command’, comments Schmitt, and he quotes from a French satirical poem in which the ultimate consequence of assigning this competence to the monarch is drawn. When the monarch determines what constitutes a miracle, the other side of the coin is that the miracles stop at the state’s behest. The monarch, *vicarious Dei*, is here elevated above God, for as Saint-Médard says in his poem ‘*de par le Roi defense à Dieu, de faire miracle en ce lieu’* (Schmitt 1938). Here the moment of ridiculousness in Hobbes’ model of state comes to the fore—in our time, not dissimilar to Saddam Hussein’s laughable declaration to the Iraqi people that Iraq had in fact won the 1991 Gulf War. Here all is political decision and command, inclusive the truth, which now finds itself ‘on the other side’ of the regime.

Thus, according to Schmitt, already at the time of Hobbes and Spinoza the criticism of hypocrisy and a merely external expression of allegiance to political power implied a certain religious primacy of the inner over the outer, of personal *fides* over official *confessio*, of piety and *vericitas* over the divine rights of the state. Divine right becomes merely the decor of a state which in reality has become a legality machine. Yes, says Schmitt, ‘whoever in general concerns himself with the distinction between inner and outer, has thereby already recognized the ultimate superiority of the inner over the outer, the invisible over the visible, the quiet over the noisy, the hereafter over the here.’^4^ Guaranteeing peace has become the state’s highest purpose in the modern era, but in this it can only succeed if its citizens refrain from opposing it with their own (religious) truths; the separation of *fides* and *confessio* is therefore also a political necessity. Genealogically speaking, here one may situate the birth of religious subjectivism and the trivializing of religion, of which more in a moment.

## II The Triumph of Subjectivism as Suspension of Tolerance

We can now take the giant step from the one extreme of what Paul Ricoeur has termed the ‘tolerance curve’—the minimal tolerance of the absolute monarchy, its obsolescence even in the totalitarian state—to the other extreme: an attitude which he summarizes as ‘*vive la différence*’.^5^ On the basis of his definition of tolerance (‘the fruit of an ascetism in the exercise of power’) Ricoeur distinguished five stages in his curve from minimal to maximal tolerance. In the first stage, I endure against my will that which I disapprove, forced by a third party arbitrator. Ricoeur’s example here is the Edict of Nantes, where two Christian confessions were forced to cohabitate. In the second stage I disapprove your manner of thinking, but at the same time I ‘make an effort to understand the manner of thinking, acting and living, finally a conception of good other than our very own.’^6^ The French philosopher is talking here about a schism or a ‘rift’ that has its seat in the individual—as an example he refers to individuals or small communities following an Erasmus, a Mélanchton, or a Leibniz. The determining stage in Ricoeur’s curve is the acceptance of pluralism in stage three, where I no longer tolerate the other on the basis of what I consider *truth*, but on the basis of the recognition of the *equal right* of the other to lead the life he wants to, the *equa libertas* in other words. In this case, my attitude is still ‘judgemental’ (as in Michael Sandel’s ‘Judgemental Toleration’),^7^ but I accept practices I morally reject because of the higher good of shared citizenship and civic peace. This dissociation of truth and justice enables many basic freedoms, to start with, the freedom of religion. The peak of the virtue Ricoeur calls tolerance is reached in stage four. Here tolerance starts losing its simultaneously passive and polemical character, because I now try to overcome the disjunction between truth and justice by allowing my own truth to be doubted and—face to face with the other’s convictions—to suspend the question of truth. Tolerance thus here not only affects the tendency to compel or coerce, but also the truth itself; I now stand open to the possibility that (political or religious) truth may exist elsewhere.

The most intriguing question raised by Ricoeur’s tolerance curve is however the fact that he also discerns a fifth stage, one where the curve starts displaying a downward trend. The demanding ‘ascetic’ curiosity with regard to the truth of the other has now changed over into a comfortable and self-sufficient *vive la différence*, delimited only by the harm principle. Here, I ‘approve of all ways of life, as long as they do not manifestly harm, third parties’.^8^ We can say that tolerance in this stage loses its ‘ascetic’ character and in a certain sense becomes superfluous as a virtue.

This is the more recent incarnation—one reaching its climax in especially the Western Europe of the past few decades—of a tolerance which had become superfluous due to the fact that religious and even moral truths have become utterly subjective, relegated to strictly private matters. *Freedom of opinion*, to Spinoza still a means of furthering the public cause as we saw, and bespeaking of faithfulness to God,^9^ now turns into the *freedom to express* any arbitrary inner—and therefore per definition ‘authentic’—upwelling or message.

In a recent essay on tolerance, the great Italian writer Claudio Magris gives an account of a trip to the Netherlands in 1988. During his trip, he attended a festival where, for all practical purposes, a boundless, permissive tolerance seemed to hold sway:

‘Innumerable stands, stalls, booths and tents, all cheek by jowl, presenting, propagating, preaching and disseminating their gospels and diverse high roads to salvation. Political parties, churches, societies, clubs, streamings and groupings all displaying their different and often totally contradictory recipes for spiritual, physical, social, metaphysical, sexual, cultural or gastronomic salvation; each had something to say and a message to proclaim: anti-militarists, veterans, and health apostles next to adherents of culinary diets or erotic techniques, esoteric cults or gymnastic exercises, ascetic or orgiastic practices; advocates of collectivization next to representatives of a wild-anarchic individualism (Magris 2013).’

Magris’ description of the Dutch festival comes close to what Ricoeur has in mind in his stage five. From his phrasings it is clear that also to Ricoeur this remarkable transition from stage four to the apotheosis of stage five raises the most questions. Does not tolerance already exceed a critical threshold in stage four, causing it to change over into something different to the virtue of tolerance? Does not perhaps a secret kinship and complicity exist between the attitudes of the last two stages? After all, ‘(…) nothing resembles more the sentence “There is also truth other than with me”, than the sentence: “Differences are indifferent”’.^10^ This sliding into indifference is responsible for the references in the title of Ricoeur’s essay to an exhaustion or ‘Erosion’ of tolerance, and to the need for a ‘Resistance’ by means of delimiting tolerance with ‘the Intolerable’.

Ricoeur also consciously describes the attitude of the fourth stage in the first person singular and in a Christian theological vocabulary, that is, as a possibility of his own Christian religion:

‘And what if, saying to myself, my conviction was not equal to the Truth (with a capital T)? After all, I don’t have the truth; I only hope (and I remind myself here of my master Gabriel Marcel) to be in the truth. All human understanding (I would add in my heart) is limited, and so also that within which ineluctably expresses my conviction. Is this not itself the destiny *par excellence* of a conviction which touches the Absolute by some side? “I am who I am”, says the God of Exodus, escaping thus the capture of literary genres in which his relation to men let itself be inscribed: stories, legislations, prophesies, hymns, words of wisdom, etc. And if I add that it is in a circular relation that a religious community recognizes itself as founded in Writings of which it has in exchange delimited the code and transmitted through the centuries the major historical interpretations, must I not conclude that this founding word in regards to my community is both supreme (in the sense that it is subordinated to nothing at all that is superior in its own meaning space) and inexhaustible, in the sense that a gap deepens between the origin of its donation and the history of its reception and its transmission? If it is indeed as such, must I not have to admit that there is also *some* truth other than for me?’^11^

The ascetism in question could thus according to Ricoeur only be phrased in the I-form, more precisely: it concerns the individual ‘in the Kierkegaardian sense, in other words, anti-Hegelian.’^12^ This is the rare ascetism of a few sages form the world’s religions, and we should, suspects Ricoeur, come to terms with the enduring gap between wisdom and citizenship of stage three. The fact that in his *Phenomenology of Spirit* Hegel deals with ‘scepticism’ after ‘stoicism’ already suggests a potential kinship: is someone who is willing to suspend his truth not already well underway towards scepticism and indifference?

The fact that in stage five ‘the differences become indifferent’ is, according to contemporary opponents of this type of tolerance, due to the ‘multi-culturalists’, whom in their texts they castigate for their cultural relativism. But this cultural relativism may have had its origins in the era of the devastating European wars of religion of the sixteenth and seventeenth centuries, in other words, at the very beginning of the modern history of tolerance and its philosophical representation in thinkers like Hobbes, Locke and Spinoza.

Like Ricoeur, Stanley Fish, and even earlier Leszek Kolakowski, have put a finger on this ‘wearing out’ or ‘erosion’ of tolerance. To say that it is preferable to tolerate the gravest of errors rather than to risk a civil war is, according to Kolakowski, culturally and psychologically ‘something completely different’ to saying that we should tolerate the most widely divergent religious convictions because differences don’t matter anyhow. Still, according to him, this second conclusion has often been drawn, and, in our own ‘hedonistic culture’, has in the meantime become common currency (Kolakowski 1999).^13^ And Fish, in his brilliant essay on Locke’s *Letter Concerning Toleration* (1689), shows that already in this text there is mention of a certain sliding of religious tolerance towards the trivialization of the religious:

‘If you say that religious belief is not something you should be rewarded or penalized for, and certainly not something you should fight about, it is only a short step to saying (what many came to say) that it is not *worth* fighting about. There is a very fine line, and sometimes no line at all, between removing religion from the public battlefield (as Locke proposed, TdW) and retiring it to the sidelines, where it is displayed only on ceremonial occasions marked by the pomp and circumstance we often accord to something we have trivialized.’^14^

In the end the religious person is expected to view his religion as ‘either something he must keep to himself or something he must offer with a diffidence that might characterize his offer of canapés at a cocktail party.’^15^

During the last few decades we in Europe have indeed become accustomed to regard religion and religious identity as the inalienable private choice and self-creation of the individual; even institutional religion’s traditional frameworks—such as churches and the passing down of doctrine—we now quickly tend to associate with the curtailment of freedom and intolerance.^16^ Even to the most enthusiastic believer, Marcel Gauchet writes with regard to France in his book *La religion dans la Démocratie* (1998), the whole idea of a nexus between our political order and religion has become an inconceivable, if not often blasphemous, thought (Gauchet 1998). The privatization of religion has now reached its completion.

### From Omnipresent to Polemical Tolerance: The Case of the Netherlands

Well now, the privatization of religion, culture and even morality could provide the basis for two completely different forms of social practice. One could hold that religious and cultural identities have an a priori right to be left in peace, to be ‘respected’ (putting it in grander terms), because, like it or not, they are expressions of self-determination. This - in the name of the virtue of tolerance—has generated a new form of hypocrisy, the culture of ‘political correctness’. Negative judgement regarding the deviant culture or religion of the other then not only becomes superfluous (religion is no longer an object of tolerance in a state where religious freedom exists), but soon after undesirable (it threatens to disrupt social cohesion—it is even considered a form of ‘discrimination’ in a number of European countries).

Multiculturalism then boils down to a cult of contact-shunning, of an enforced silence amongst various subcultures and population groups. And indeed: once my religion or culture has become my very personal creation or *design*, then nothing needs to be ‘ascetically’ tolerated anymore, for nothing is being shared or strives towards public recognition, and thus nothing risks raising contradictions or irritation any longer. One could also say that a single notion regarding the status of religion, culture and even political conviction has gained complete dominance: these are mere means to self-realization, personal hobbies even. The notion that we are primarily concerned with our own ‘self-realization’ and self-expression, then has become so prevalent that we are hardly able to conceive of commitments to political or ethical ideals or faithfulness to a religious conviction as anything but loincloths covering up emotional needs, peculiarities of character or private ambition. Hence the media’s untiring hunt for ‘the man behind the politician’.

But this form of tolerance could just as well generate an utterly polemical form of intolerance. The surprising step from ultra-tolerance to intolerance was put into words by the German philosopher Robert Spaemann as follows. The demand to respect other convictions, he writes, ‘then changes over into the demand to have no convictions, on the basis of which one then holds opposing views as incorrect (Spaemann 2002).’ Tolerance itself now becomes indicative of a political line separating an ‘us’, the tolerant part of the world, from a ‘them’, the bearers of worn-out, backward and intolerant traditions. Claudio Magris formulates the transition from a tolerance-become-absolute to intolerance even sharper: ‘The statement ascribed to Voltaire, that he was prepared to rise in mortal defence of his worst enemy’s right to free expression, finds its ironic historical counterpart in those who are so passionately tolerant, that they are prepared to put all intolerant people up against the wall.’ 17

In the Netherlands and elsewhere in Europe, a tolerance turned polemical has precipitated an outcome which is the complete opposite of political correctness. The expression of authentic personal feelings, judgements and prejudices has become sacrosanct, and any discussion concerning boundaries is dismissed as an invasion of freedom or act of moralizing. Ian Buruma, author of an illuminating book on the recent political and social turbulence in the Netherlands, rightly perceives a certain kinship between the unrestrained verbal self-expression of the murdered Dutch film director and Islam critic, Theo van Gogh, and the devoutness of the Dutch pietistic tradition, where inner feelings are imbued with a sheen of holiness, and considered superior to all external cultures and cults—which, as we have seen, Carl Schmitt sees already starting with Hobbes:

‘The insistence on total frankness, the idea that tact is a form of hypocrisy, and that everything, no matter how sensitive, should be stated openly, with no holds barred, the elevation of bluntness to a kind of moral ideal: this wilful lack of delicacy is a common trait in Dutch behaviour. Perhaps its roots are in Protestant pietism, a reaction to what was seen as glib Catholic hypocrisy. Private confession had to become public. Discretion was a sign of holding back the truth, of dishonesty (Buruma 2007).’

Ian Buruma was also right, when he discerned a like-mindedness between van Gogh and the editorship of *Charlie Hebdo* who were assassinated by terrorists in Paris in January 2015 (Buruma 2015). So the absolute tolerance has triggered the heinous practices of religious gangsters.

## III Fundamentalism as Remedy: Isaac Bashevis Singer’s The Penitent

By means of a somewhat different route also Robert Spaemann lands up at the issue of subjectivism as suspension of tolerance, *and* that of ‘fundamentalism’ as extreme but *modern* response to it. When Ricoeur, as we have seen, at the peak of his curve acknowledges the ‘circular’—and therefore contestable—nature of the link between his religious community and her foundation in Holy Scripture, he is in fact pointing out the well-known problem of the hermeneutic circle. Spaemann does the same in an essay on the modern phenomenon of fundamentalism. As is well known, in its initial phase Protestantism returned to Scripture out of the conviction that the stream emanating from the source (tradition) had insufficient authority, and the institution of interpretation binding tradition to the source (the Church) had become incompetent and perverted. ‘*Das Wort sie sollen lassen stahn*’, wrote Luther in defence of the letter of the text, against dogmatic appropriations, and in order to curtail free investigations of Scripture by the solitary individual. Which of course did not allow him to escape the hermeneutic circle either. For even if the text is the final criterion, the text is always given to us as interpreted text, for the act of reading is already one of interpretation.

Well now, argues Spaemann, an element of the abyss lurks in this hermeneutic circle, for it ‘seems to open the door to arbitrariness, and to make everything, including atheism, compatible with the Bible (Spaemann 1989).’ (Protestant) fundamentalism which wants to interpret Scripture literally, then becomes a kind of self-deception in order not to face the real problem—hermeneutic subjectivism, arbitrariness -, while the Catholic church’s answer to the problem has been to establish a formal monopoly on interpretation.

Mildly put, historically seen neither solution has been without its problems, and one should probably conclude that in both instances the arbitrary moment in the hermeneutic circle had merely shifted. Even today still, expressions of solo investigations into Scripture by believers and theologians alike regularly sees the Catholic church feeling itself forced to react with repressive measures, and the discrepancy between lip-service to Rome’s religious truth and actual practice (in short: hypocrisy) has to many become synonymous with the adjective ‘catholic’. In Protestant churches on the other hand, orthodoxy has always enjoyed a rather precarious status, and subjectivistic enrapturement and fundamentalism have been recurring problems. Of the fact that subjectivistic relativism and ultra-orthodoxy call forth and hold one another in a grip, the Jewish novelist Isaac Bashevis Singer (1904–1991) gives a literary account in *The Penitent* (1983), the novel which first clarified to me the central impulse in the psycho-moral genesis of the spiritual attitude we nowadays refer to as fundamentalism.

*The Penitant* is a first person account of the life of Joseph Shapiro, a Polish Jew who witnessed the German bombing of Warsaw in 1939, survived the war, and in 1947 emigrated to the United States with his girlfriend. Told retrospectively, Shapiro’s story is of the deep personal and existential crisis he suffers in his adoptive country, and of his radical decision to start a new life in an ultra-orthodox Jewish community in Israel. He describes his growing repugnance with his life in a left-liberal environment under modern and late-capitalist conditions. I summarize a number of elements from his description.

As a dealer in real estate he made *big money*, yet at the same time became increasingly haunted by the thought that by itself, making money was utterly meaningless. In his own circle, adultery was regarded as a supreme virtue. Conjugal fidelity was tantamount to ‘constantly eating the same dish’, held his friends (Singer 1986). Also he and his wife—the marriage remained childless—both take lovers. But a man living with different women quickly becomes an expert in telling lies, and the fact that one of his mistresses was starting to clean him out financially, left him feeling that her love had been bought. They frequently visit the theatre and films in which obscenities, extreme violence, scheming and deceit are portrayed as the most normal things on earth. They read highly fashionable authors and Western thinkers, but he often caught himself wondering whether they actually had anything to say. Often the authors’ message would simply read: we live in an abattoir, and there’s nothing to be done about it. And the labour of Western philosophers would essentially seem to boil down to saying that with regard to what really matters, we know nothing, and can know nothing.

The American legal system left him with the impression that paying bribes to police and judges was quite commonplace, just as it was for the legal profession to ensure murderers and rapists remain out of prison. Disrespect of the elderly was considered normal; this was the reason why everyone was colouring their hair, having nose jobs or make-overs, why he heard elderly people saying they were ‘80 years young’. Many turned to therapy, and especially psycho-analytic therapy always seemed to have the same answer: it is someone else’s fault. Your mother was too dominant, while your father on the other hand was too often absent or too insensitive. However, in the circles in which he moved, to criticise such practices was regarded as meddling in the affairs of others, simply *not done*. In the meantime, his leftist friends and acquaintances had posters of Lenin and Stalin on their walls, and were dreaming of a communist revolution and the wrath of the masses. To him, reading the paper felt like swallowing a daily dose of poison: war, murder, rape and deceit.

Also the structural cruelty towards animals he found increasingly difficult to stomach. The thought that ‘where animals are concerned, we are all Nazis’, took root in him. In my opinion, this is the key sentence in the novel, for the thought of ultimately not being far removed from the Nazis, completes his crisis. ‘My whole being was one skein of bitterness, sourness and shame over my own degradation.’^18^ ‘Run from the culture of Hitler and Stalin!’, Shapiro concluded: I must return to my Jewish roots.^19^ From this moment on, he felt ‘like a beast (running) from a forest fire’.^20^ The key term here is *separation* from all ‘worldliness’: worldly clothing, worldly women, worldly idols. After much hesitation and self-doubt (wasn’t he merely being the victim of some new form of self-deception?) he makes a radical break with his life in the United States, with his wife, his mistress, his friends and his work, and he gets on a plane to join an ultra-orthodox Jewish group in Israel. ‘I let my beard and hair grow, donned an upper garment, and broke completely with all things associated with modern Judaism’. Here any compromise must be strongly rejected, for assimilation is adaptation, and adaptation opens the way to Nazism and therefore a radical unfaithfulness towards his ancestors.

*Faithfulness* and *unfaithfulness* is indeed the central and completely binary opposition in this account of conversion, for the ‘separation’ which it necessitates is in service of a representation of integrity and purity, as represented by the ancestors. Conversion to a Jewish-orthodox way of life here takes the form of a break with ingratitude. This ingratitude Shapiro experiences in such a profound way that he associates it with the practices of his parents’ henchmen.

Of course it is possible to dismiss this very dark sketch of 1970’s American society and Shapiro’s environment as a gnostic caricature. And the opinions of recent converts (irrespective of to or from a religion) should naturally be taken with a pinch of salt. But by leaving it at that we would probably be missing the disquieting and ‘dangerous’ aspect of this novel. In their study *Occidentalism* Avishai Margalit and Ian Buruma give many examples of the birth of fundamentalism (and terrorism) out of a ‘romantic’ aversion against modern life and modern culture, and this as well within the West itself as by non-western people who were living for a period in a western country, as for example Sayyid Qutb, born in Egypt, who lived for e period in New York; out of his disgust of this city was born one of the most influential Islamist thinkers of the twentieth century (Buruma and Margalit 2004).

Gratitude and faithfulness, as well as their opposites, refer to attitudes regarding our parents and other ancestors, to their legacy. But hidden underneath the often ostentatious posing of religious fundamentalism lies the question of how traditions could come to accept change, even understand it as progress. This would necessitate a hermeneutic labour of developing criteria both for safeguarding continuity with the origin and with tradition, as well as to distinguish improvements from things which are likely to detract.^21^

During the past decade the need for this has been dramatically evident with regard to Muslim immigrants in Europe. Let me give an example here. A while back in France, the European Muslim intellectual Tariq Ramadan proposed a moratorium on the practices of corporal punishment prescribed in Sharia law with regard to certain ‘offences against God’ (Roy 2005). For this he was severely criticized by French secularists, who saw in this proof of his hypocrisy and his ‘forked tongue’. After all, Ramadan did not propose simply abolishing these practices altogether, and his allegiance to the French Republic could therefore be considered only half-hearted and ambiguous. Unjustly so, argues the Islam scholar Olivier Roy, for with this Ramadan in fact gave theological recognition to the secular state and its monopoly on penal practice, while at the same time introducing a kind of Islamic purgatory. This is in fact the kind of much-needed hermeneutic labour required to make it possible for a Muslim citizen to be part of a secular state.

## IV The Testing of the Democratic Separation Between Truth and Justice in Our Time

Gratitude and faithfulness, as well as the hermeneutic circle or the unfaithful element woven into all faithful practices, is not a problem which only concerns Muslims. In a country like the Netherlands, the prevailing political tendency of taking the most recent liberal consensus as unshakable point of departure with regard to the integration of immigrants, in fact testifies to a repression of our own—in part religiously coloured—history. A Dutch politician already pointed this out in 2006, when he wrote that, while a politicised Islam in the Netherlands could indeed prove troublesome, he at the same time had the strong impression that, conversely, the Dutch were busy ‘turning (their) political consensus into a religion’. One of the examples he gave concerned the official Dutch naturalization film shown abroad to potential immigrants: ‘The film suggests that, as a Muslim, one would have to accept things like Amsterdam’s Gay Parade and the likelihood of encountering topless girls on beaches. Should this indeed be the condition for being a Dutch citizen, then we might as well—retro-actively—deny it to our own parents. For what they in their day rejected, is of course precisely that which now *has* to be tolerated (Wöltgens 2006).’ In other words, a recent, contingent liberal opinion here becomes fixed as norm and truth, as component of the national identity. In 2013, a Dutch Social-Democrat minister made known that he considered not only enforcing obedience to Dutch laws, but also the ‘internalization’ of Dutch morality—and thus for example also a ‘positive attitude’ towards homosexuality—as duties of government.^22^ This marks a step in the direction of a new truth-regime, one which does not accept the disjunction between truth and majority decisions; however, undoubtedly also this time the net result would largely be hypocrisy.

Another example of this tendency towards establishing truth-regimes was given in 2006 by a specialist on Turkey, E.J. Zürcher, in connection with Turkey’s potential entry into the European Union—a topic of considerable importance not only to Europe, but also in geopolitical terms. He pointed out a remarkable paradox: to the very extent that European politicians tend to outright dismiss the prospect of Turkish membership, their own societies are starting to resemble that of Turkey. Not 10 years ago, he established, Turkey was reproached for being insufficiently democratic, insufficiently secular, and overly nationalistic—besides for refusing the blessings of a multi-cultural society, and for oppressing its ethnic minorities. For in Turkey, of everyone it was expected to repeat after Atatürk: ‘fortunate is he who can say: “I am a Turk”’.

However, in the Netherlands of today the ideal of the multi-cultural society has already been laid to rest by all major political parties. National sovereignty is again being played out polemically against Europe, and formulating a national identity which could be held up to newcomers is—as we have already seen—regarded as an urgent task by many. Meanwhile most of the Dutch population has become willing to unquestioningly sacrifice liberal freedoms on the altar of the fight against terrorism (Zürcher 2006).

Most spectacular is the symmetrical stalemate reached with regard to the question of Turkey’s official recognition of the 1915 Armenian genocide. While France has recently adopted a law making *denial* of the 1915 genocide a criminal offence, Turkey knows a much older law with which to gag those holding deviant opinions (like for instance *acknowledgment* of the 1915 genocide). This affects the essence of tolerance as an institutional characteristic of liberal democracy, the disjunction between truth and justice given in the third stage of Ricoeur’s tolerance curve. Here we need to take heed of what Charles Taylor calls ‘state neutrality’. This entails ‘avoid(ing) favouring or disfavouring not just religious positions but any basis position, religious or non-religious (Taylor 2011).’ And the democratic state should also refrain from laying down its own version of historical truth.

## Conclusion: Existential Versus Political Fundamentalism

I end with a conclusion regarding fundamentalism and the threat it poses to the institutional tolerance of liberal democracy. In his essay on (Christian and Muslim) fundamentalism, Robert Spaemann advances the thesis that the ‘dangerousness’ of a religion or life conviction ‘is nothing but the obverse of the gravity’ these hold to their adherents.^23^ As I have previously argued, I tend to agree with Spaemann on this, and even think that it holds true for those who have elevated ‘tolerance’ to a supreme value: then also this virtue of restraint and openness could become ‘dangerous’, even violent. In both instances, a certain putting into perspective of this gravity is required to prevent them from assuming their grimmer forms. Only greater self-consciousness could for instance grant Muslims and Islamic peoples the creativity required in their future grappling with the hermeneutic circle, and the equanimity to bear the narcissistic wounding given with any passionate quest for truth in modern democracies. After all, democracy by definition demands a restriction or ‘circumcision’ of our ‘truths’, in as far as they are always qualified by democratic majorities, able to promulgate and change laws.

In the foregoing I further suggested that the danger of fundamentalism also lurks in the tendency—undeniably present in Europe—to fight fanaticism *fanatically*, in other words, by introducing elements of an authoritarian truth-regime to democracy. Then, as of old, it turns into a clash of opposing fanaticisms. In contrast to the so-called hermeneutic civil wars of sixteenth and seventeenth Europe, the battle lines are now not drawn between opposing religions, but rather between religious and non-religious or anti-religious parties – as was demonstrated by the Danish cartoon incident and, more recently, by the attacks at the beginning of 2015 on *Charlie Hebdo* and on the cultural centre in Copenhagen where freedom of opinion in a secular state was the topic of the discussion. Public debate regarding both the place of Islam in Europe and religiously-founded terrorism does indeed frequently threaten to sink into a debate essentially on religious belief versus (enlightened) atheism. In the one corner *in extremo* believers, who, along with Dostoyevsky (and Joseph Shapiro)^24^ affirm that ‘if God does not exist, everything is permitted’, and in the other, certain non-believers maintaining that ‘precisely should God exist, everything would be permissible’.

But we must reject this opposition. I would even state that only by acknowledging the aspect of truth in both statements and by taking as its point of departure the normative principle held in common by both positions (‘not everything should be permitted’), does a pluralistic democratic society become a real possibility. In order to affirm this aspect of truth in Dostoyevsky’s statement, a non-polemical definition of the term fundamentalism is required, as for instance that proposed by Spaemann and in Singer’s novel. A fundamentalist in this sense would be ‘someone to whom something is sacred, to the extent that he is unwilling to put it up for discussion.’ Departing from this definition, each and every normal person would be a fundamentalist, for someone to whom nothing is sacred is capable of anything. ‘God’ in Dostoyevsky’s statement then signifies the sacred which enables co-existence, while the interpretations of it are bound to forever remain subject to and broken by democratic decisions. Spaemann calls this an existential fundamentalism, in distinction to a political fundamentalism which he analyses as a form of totalitarianism for its making absolute the political perspective: its judging of each and every aspect of life according to its positive or negative functionality to the state.^25^

But the atheist also has a point. To the terrorist executing ‘God’s will’ (sometimes not excluding acts of summary execution as we experience today, for example by *ISIS* or *Boko Haram*), the pretence of having a hotline to God (or a secular substitute, like communism’s ‘historic necessity’) of course justifies sweeping aside all ‘mere human’ scruples and considerations. Against this, Slavoj Žižek defends the dignity of atheism, which has to make do without the will of God and the promise of divine reckoning. His example is however drawn from the Christian universe. At the time of Louis IX’s crusades, the chronicler Yves le Breton reported of an encounter with an old woman carrying a bowl of fire in her left hand and one of water in her right. When asked why she was carrying the bowls, she replied that with the left she wanted to set paradise ablaze, and with the right, extinguish the flames of hell. For, she added, ‘I do not want anyone to do good on account of promise of paradise or the fear of hell, but solely for the love of God’. Nowadays, adds Žižek, it would seem that ‘this authentically Christian ethical position has survived mainly within atheism’.^26^

## Footnotes

1 Walzer, o.c., XII.

2 See de Wit (2010)

3 Spinoza, *Theological-Political Treatise*, 257.

4 Schmitt, o.c. 95.

5 Paul Ricoeur, ‘L’usure de la tolérance et la résistance de l’intolerable’, in: *Diogène* no. 176, oct.-dec. 1996, 166–176; my quotes are from the Engelish translation: (Ricoeur 1996)

6 Ricoeur, o.c., 192.

7 (Sandel 1996). Judgemental Toleration ‘assesses the moral worth or permissibility of the practice at issue, and permits or restricts it according to the weight of these moral considerations in relation to competing moral and practical considerations.’(107) In the examples he gives it is clear that the toleration of some ‘evil’ is desirable when a greater good is made possible or a greater evil is avoided by its permission. See for a similar viewpoint my ‘Why Tolerance cannot be our Principal Value’, in: *Bijdragen. International Journal in Philosophy and Theology* 71/4, 2010, 377–390.

8 Ricoeur, o.c., 191.

9 Spinoza,o.c. 259: ‘And if we, in conclusion, would also give consideration to this, that the loyalty of the individual to the public cause and God alike could only be known from his works, namely the love of his neighbour, we would have no doubt that the best form of government would permit the individual the same freedom to philosophize which, as we have earlier demonstrated, also religion permits.’

10 Ricoeur, o.c., 197.

11 Ricoeur, o.c., 194–195.

12 Ricoeur, o.c, 195.

13 (Kolakowski 1999); see also (Kolakowski 1982)

14 Fish, o.c., 175.

15 Fish, o.c., 59.

16 See Jonkers (2002).

17 Magris, o.c. 10.

18 Singer, o.c. 27.

19 Singer, o.c., 50.

20 Singer, o.c., 85.

21 Spaemann, o.c. 47.

22 For my critical response see: (de Wit 2013).

23 Spaemann, o.c. 47.

24 Singer, o.c., 117: ‘There’s no such thing as morality without religion. If you don’t serve one idol, you serve another.’

25 Spaemann, ‘Versuch über den Fundamentalismus’, 48.

26 Slavoj Zizek, ‘Bluttrübe Zeiten. Die Antinomien der toleranten Vernunft und die Würde des Atheismus’, in: *Lettre International* 73, Summer 2006, 10–17; 14.
